# Insights of Novel Coronavirus (SARS-CoV-2) disease outbreak, management and treatment

**DOI:** 10.3934/microbiol.2020013

**Published:** 2020-07-03

**Authors:** Dharmender Kumar, Lalit Batra, Mohammad Tariq Malik

**Affiliations:** 1 Department of Biotechnology, Deenbandhu Chhotu Ram University of Science and Technology, Murthal-131039, Sonepat, Haryana India; 2 Institute for Cellular Therapeutics,Departments of Microbiology and Immunology, University of Louisville, Louisville, Kentucky-40202, USA; 3 Departments of Microbiology and Immunology, Regenerative Medicine and Stem Cell Biology.and James Graham Brown Cancer Center, University of Louisville, Louisville, Kentucky-40202, USA

**Keywords:** COVID-19, therapeutic, vaccine, pandemic, transmission, outbreak, SARS-CoV-2

## Abstract

Emerging and re-emerging viral diseases poses a threat to living organisms, and led to serious concern to humankind and public health. The last two decades, viral epidemics such as the severe acute respiratory syndrome (SARS-CoV) reported in the years 2002–2003, and H1N1 influenza (Swine flu) in 2009, middle east respiratory syndrome (MERS-CoV) from Saudi Arabia in 2012, Ebola virus in 2014–2016, and Zika virus in 2015. The recent outbreak of 2019-CoV-2 or severe acute respiratory syndrome-2 (SARS-CoV-2), novel coronavirus (2019-nCoV, or 2019 disease, COVID-19) in Dec 2019, from, Wuhan city of China, has severe implications of health concerns to the whole world, due to global spread and high health risk. More than 423349 deaths had occurred globally and is still increasing every day. The whole world is under a health emergency, and people are advised to stay at their homes to avoid the spread of person-to-person infection, and advised to maintain social distancing. The advancement in clinical diagnosis techniques like Real-Time PCR (RT-PCR), immunological, microscopy, and geographic information system (GIS) mapping technology helped in tacking the rapid diagnosis and tracking viral infection in a short period. In the same way, artificial intelligence (AI), combinatorial chemistry, and deep learning approaches help to find novel therapeutics in less time and wide applicability in biomedical research. National Institute of Allergy and Infectious Diseases (NIAID) has started the clinical trials of investigation COVID-19 vaccine. Therefore, we can expect vaccines to be available for this deadly disease in the coming few months.

## 1. Introduction

Due to the outbreak of SARS-CoV-2 from Wuhan, China, and its spread to the different countries. The global health emergency has declared by the World Health Organization (WHO) to prevent its spread. The COVID-19 disease has clinical signs ranging from mild respiratory illness to pneumonia and severe consequences in terms of high health concerns, now the whole world is on at high health risk alert [1]. Understanding the role of civet in severe acute respiratory syndrome (SARS-CoV), and camel in middle east respiratory syndrome (MERS), an outbreak of Ebola in Central Africa [2]. This provides the avenues in terms of pathogenesis, design of diagnostic, antiviral, and vaccine to control high risk emerging infection [3]. Further, the study published in Lancet 2020, classified this virus in the diverse group from SARS-CoV, a new human-infecting subgenus *Sarbecovirus* of the genus *beta*-*coronaviridae*, and genetically distinct from SARS-CoV (79%) and MERS-CoV (50%). Phylogenetic analysis has shown that bats might be the original host and exhibited 88% genome similarity with bat viruses, bat-SL-CoVZC45, and bat-SL-CoVZXC21. Further sequence feature and analysis indicated that SARS-CoV-2 may bind to the angiotensin-converting enzyme-2 receptors of humans [4]. However, public health authorities are monitoring the situation very carefully to understand the new virus outbreak. Wang et al. (2020) studied the clinical, genetic, and epidemiological features of COVID-19. By comparison of SARS and MERS past outbreaks, it was shown that the total fatality rate of COVID-19 is about 3.4% as per the reports based on CDC data. The genomic comparison of the sequenced strains of the virus has indicated to device the necessary control measures for the spread of the disease and standards for the diagnosis and potential interventions required. Within the period of last 17 years, there has been the emergence of two new SARS virus (CoV and SARS-CoV-2) which creates attention to think about the reasons for the development of new strains [5] and evolution of new novel virus can be modeled through modification of the virus to avoid their risk to human transmission [6].

The outbreak is still increasing with the fatal consequences to humans due to their rapid spread through person-to-person transfer and entering international countries. The travel risk of the virus leads to its faster spread of this disease [7],[8] and has further consequences in terms of regional and global range [9]. The preliminary transmission of basic reproduction of nCoV-2 from 2019 to 2020 estimated using basic reproduction number, (R0 or reproduction number defined as the number of additional cases produced due to single infectious case in disease susceptible population) based on the data received from the early phase of this outbreak; this was due to non-availability real-time data at that time. The mean R0 of COVID-19 ranges from 2.24 to 3.58, indicating the outbreak nature of this virus [10]. WHO has declared COVID-19 as an international pandemic, the total confirmed cases of COVID-19 infections globally as 7553182 confirmed cases with 423349 deaths and there has been a continuous upsurge in the data (WHO, COVID-19 situation reports-145, June 13, 2020).

Clinical studies had indicated that chloroquine phosphate efficacy for the treatment of COVID-19 disease affected *Pneumonia*. This drug has been included in the multicenter clinical trials in China [11] and been approved by the FDA for US patients. This drug been used for the treatment of Malaria as an anti-inflammatory agent and used to treat rheumatoid arthritis and systemic lupus erythematosus (SLE). The studies have shown that its antiviral activities due to an increase in endosomal pH, which is required during viral infection and cell fusion. The drug has been shown to interfere with glycosylation of cell receptors of SARS-CoV [12] and effective in the treatment of avian influenza A H5N1 [13]. This review was prepared to understand the outbreak of COVID-19, transmission between the population, and treatment options available, and future scope in the area. The publish papers have indicated the world health risk of COVID-19 virus as per the WHO and indicated the international pandemic nature of COVID-19 with severe epidemiology and pathogenesis [14]–[16]. The advancement in detection method and geographic information system (GIS) mapping technology based tracking has helped in the rapid spread analysis of COVID-19 cases. Such data has been updated on various online resources [17] (Table 1).

## 2. Data used

The PubMed, Medline and other online resources searched for the articles describing novel coronavirus, COVID-19, control and treatment, etc search options. The data on the latest research is from the Centre for Disease Control, available from http://www.cdc.gov, and the recent research update on NIH available from https://www.nih.gov/ and other web resources were consulted for this paper (Table 1). NCBI: severe acute respiratory syndrome-2 database searched, retrieved the useful information, and analyzed SARS-CoV-2 GenBank data. The latest data for the COVID-19 around the world was accessed from the WHO situation report.

## 3. Transmission of coronavirus

**Figure 1.**
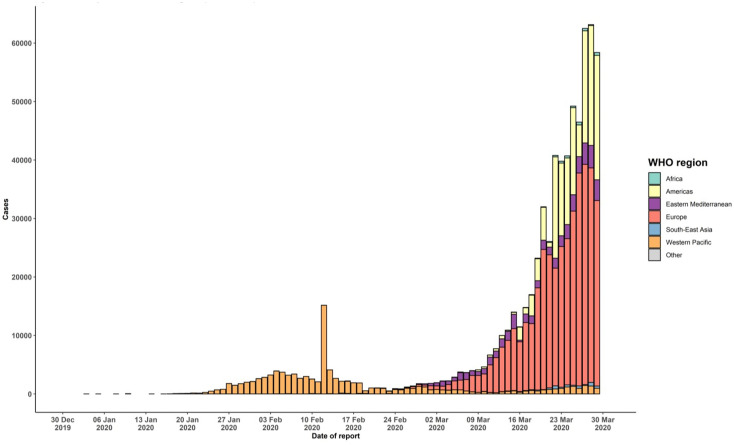
Epidemic curve of confirmed COVID-19, by date of report and WHO region through 2 April 2020 (reproduced with permission from WHO, Coronavirus disease 2019 (COVID-19) Situation Report).

The transmission of COVID-19 disease when compared to SARS and MERS is asymptomatic or less symptomatic. Similar to other viruses, this virus is also a type of enveloped virus, and its envelope protein structure was explained [18]. Human-to-human transmission is faster due to the movement of person(s) from one place to another, and such type of behavior has been observed for all forms of respiratory viruses in humans [19]. The vertical transfer occurs from unrelated species from animal to human, was due to genetic mutation and recombination of old strains of coronavirus. There is a need to block the chain of transmission by isolation and quarantine measures to stop the entry of viruses from person to person. Therefore, social distancing is the need of the hour. The surveillance pyramid has less symptomatic at the bottom line in which the ‘*patients do not seek health care, do not receive a diagnosis, and may spread the virus to contacts*,’ and then severe to fatal infection as ‘*patients seek health care and can be diagnosed and isolated, and their contacts can be traced*. This has a propensity for nosocomial spread [20]. Low temperature and humidity might provide conducive environment for re-infection of viruses as in China, and contact during social gathering leads to the rapid trajectory of epidemic spread [5], family cluster-based transmission [3], and other early transmissions in China [21]. Due to emerging infectious disease though SARS, MERS, and SARS-CoV-2, there is a risk of transmission of thorough coronavirus transfer of labile blood products [22]. The epidemic curve of confirmed cases has shown the spread to different countries, and outbreaks in the world have been displayed in the shaded area (Figure 1).

**Table 1.** The online resources for the information of coronavirus disease (COVID-19).

Sr No.	Website/ database resources	Salient features	Other Information, if any
1	https://www.who.int		
2	https://www.statnews.com		
3	https://www.viprbrc.org	Virus Pathogen Database and Analysis Resource (ViPR )	
4	https://www.ncbi.nlm.nih.gov	NCBI	
5	https://www.coronavirus.gov, www.cdc.gov and https://www.ecdc.europa.eu	CDC and European Centre for Disease Prevention and Control	
6	Get the latest research from NIH: https://www.nih.gov/coronavirus	National Institute of Health, NIH USA	
7	https://www.gisaid.org/		
8	https://www.bloomberg.com		
9	https://www.zdnet.com		
10	https://coronavirus.jhu.edu		
11	https://www.fastcompany.com/		
12	https://pages.semanticscholar.org The dataset contains all COVID-19 and coronavirus-related research (e.g. SARS, MERS, etc.) from the following sources: PubMed's PMC open access corpus using this query (COVID-19 and coronavirus research) Additional COVID-19 research articles from a corpus maintained by the WHO bioRxiv and medRxiv pre-prints using the same query as PMC (COVID-19 and coronavirus research) We also provide a comprehensive metadata file of 29,000 coronavirus and COVID-19 research articles with links to PubMed, Microsoft Academic and the WHO COVID-19 database of publications (includes articles without open access full text). Citation: When including CORD-19 data in a publication or redistribution, please cite the dataset as follows: In bibliography: COVID-19 Open Research Dataset (CORD-19). 2020. Version 2020-03-13. Retrieved from https://pages.semanticscholar.org/coronavirus-research. Accessed YYYY-MM-DD. doi:10.5281/zenodo.3715506In text: (CORD-19, 2020)	New developed Access this dataset to help with the fight against COVID-19 open Resource database CORD-19 developed by a product of collaboration between the National Institutes of Health, Microsoft, the Chan Zuckerberg Initiative, and the Allen Institute for Artificial Intelligence, among others	A Free, Open Resource for the Global Research Community Accessed through www.sementicscholar.com
13	https://ourworldindata.org	Coronavirus Disease (COVID-19) – Statistics and Research	
14	https://bigd.big.ac.cn	China National Center for Bioinformation 2019 Novel Coronavirus Resource (2019nCoVR)	
15	https://www.worldometers.info	COVID-19 CORONAVIRUS/CASESUPDATES-Case Graphs-Death Graphs-Countries-Death Rate-Incubation-Age	
16	https://firstdraftnews.org	Database for reporters	
17	http://statista.com	COVID-19 disease advice and information NIPH-FHI	
18	Resources from the Allen Institute for AI: SciSpacy, a text processing toolkit optimized for scientific text SciBERT, a BERT model pretrained on scientific text Semantic Scholar APIandOpen Research Corpus Create an AI-powered customizable adaptive feed of COVID-19 research View the latest search results for COVID-19 on Semantic Scholar		
19	Additional Resources: COVID-19 Research Database (provided by the WHO) LitCOVID (provided by the NIH) COVID-19 Resource Page (provided by Microsoft Academic) COVID-19 Research Export File (provided by Dimensions) Day-Level COVID-19 Dataset (hosted on Kaggle) COVID-19 Global Cases (provided by Johns Hopkins University) Blog Post: Computer Scientists Are Building Algorithms to Tackle COVID-19	Additional resources by various institutes and organization	
20	Publisher Resources: American Society for Microbiology BMJ Elsevier New England Journal of Medicine Springer Nature Wiley	Resources developed by publishers	

The first known case of person-to-person transfer of SARS-CoV-2 has been detected in the USA [23], Vietnam [24], and Korea, through tertiary care center [25] and other infected countries. The comprehensive sequence analysis suggests that the SARS-CoV-2 seems to have the property of both bat and an origin-unknown virus. This recombination might occur in the viral spike glycoprotein. Ji et al. have suggested that homologous recombination might be responsible for SARS-CoV-2 for its cross-species transmission [26]. It has been observed that infection transmission occurs through an infected person's ocular surface or dental structure [27]. This may happen when coming in contact directly/or indirectly, by coming in contact with saliva, blood, and other body fluids, and occurs through instruments as well [28]. The patient screening, environmental control measures, personal and equipment protection, and critical care operations should be needed for taking care of local outbreaks of SARS-CoV-2 [29]. The complete genome sequence of SARS-CoV-2 virus strain has been elucidated from Nepal person who recently returned from, Wuhan China [30] and from other countries as well and till date 170 such partial/ complete genome sequence available online in NCBI database.

Based on analysis of SARS-CoV and MERS-CoV reports on critical cases of mother illness, spontaneous abortion and may lead to her death[31]–[33]. Shek et al. reported prenatal transmission of the SARS, five live-born infants born to pregnant women having SARS infection during the outbreak in Hong Kong, none of the infants developed clinical, and other manifestation with SARS [34]. The more recent study by Yan et al., four pregnant women were confirmed to have COVID-19 infection, and the babies were healthy [35]. Similar results were reported by Assiri et al., five cases of pregnant women infected with MERS-CoV from Saudi Arabia [33], and the recent study by Hunag et al., had shown that positive test mothers can deliver the healthy baby [36].This has been demonstrated that vertical transmission of the virus did not occur in any of these cases. Whereas, the human-to-human transmission occurred through close contacts, as suggested by (Li et al.), regarding new transmission in China [9]. Deng and Peng (2020) suggested the characteristics and public health response of the COVID-19 outbreak in China and concluded, person-to-person transfer, are the primary cause of transmission. This had occurred at the time, when China is celebrating the spring festival, and at this time occasion, more peoples were gathered. However, the first report appeared in December 2019. After that, it spread at a rapid pace and get transmitted to other countries through their visitors to China. Although the mortality rate is low for COVID-19 (2.67%) as compared toSARS (9.60%), and MERS (34.4%).According toa report on the ‘*diagnosis and treatment scheme of pneumonia, (III Ed)*,’ the infection occurs through a person- to- person and the infected person with no sign of infection may act as the source of spread of disease. As reported, the virus is heat-sensitive, gets killed at 56 °C (30 min), and disinfected with ether, 75% ethanol, chlorine disinfectants like 10% bleach, peracetic acid, and chloroform.

**Figure 2.**
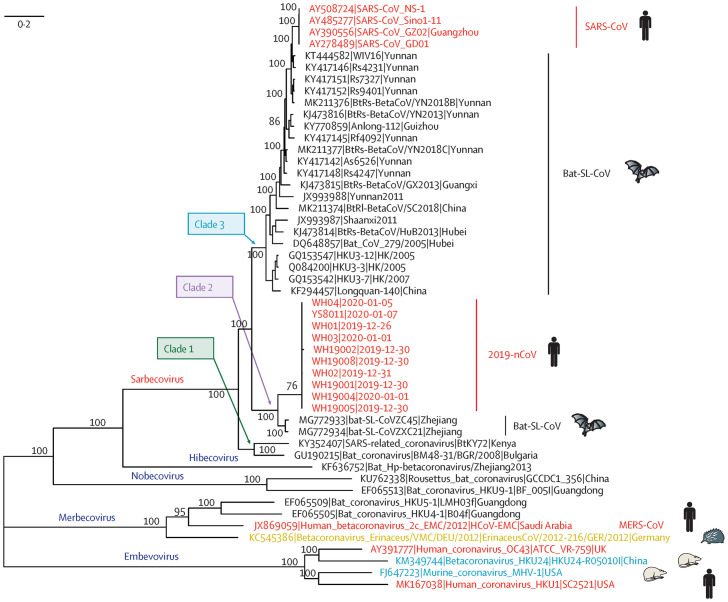
Phylogenetic analysis of full-length genomes of 2019-nCoV and representative viruses of the genus Betacornaviridae (reproduced with permission from publisher from Reference, Lu et al. 2020, Lancet. 2020 Feb 22;395(10224):565-574) [4].

**Figure 3.**
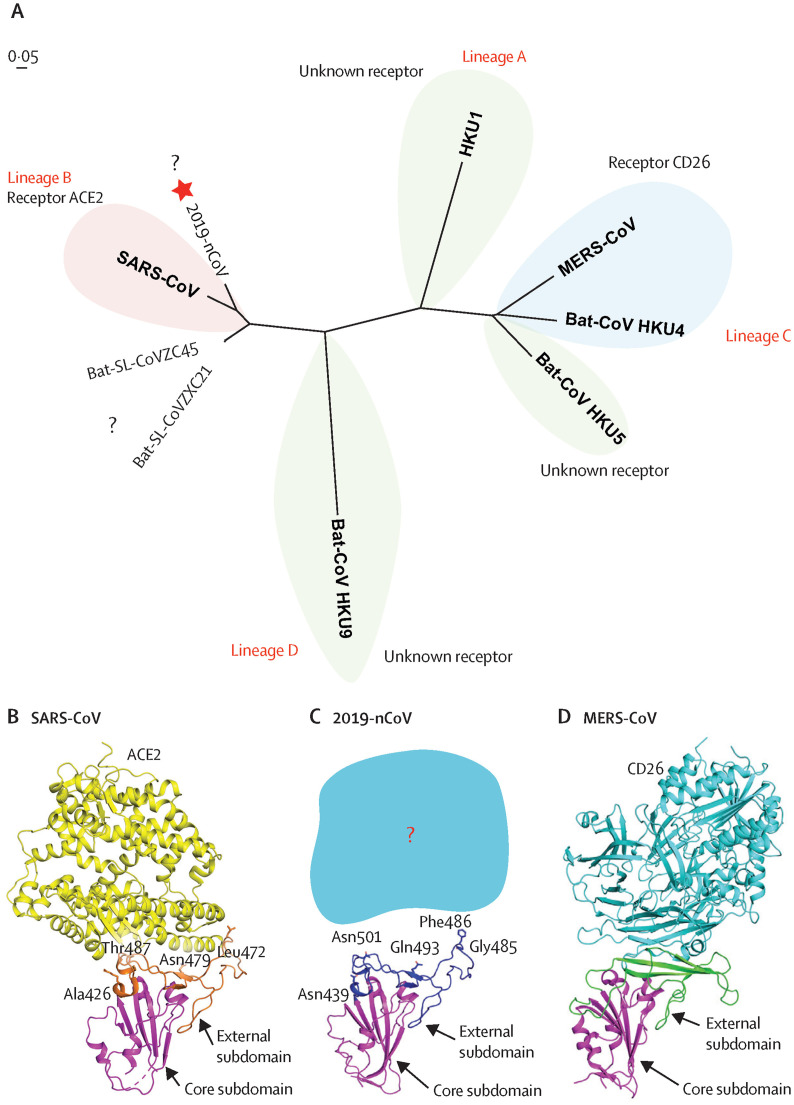
Phylogenetic analysis and homology modeling of the receptor-binding domain of the SARS-CoV-2, SARS-CoV, and MERS-CoV. (A) Phylogenetic analysis of the receptor-binding domain from various betacoronaviruses. The star highlights SARS-CoV-2 and the question marks means that the receptor used by the viruses remains unknown. Structural comparison of the receptor-binding domain of SARS-CoV. (B), 2019-nCoV (C), and MERS-CoV(D) binding to their own receptors. Core subdomains are magenta, and the external subdomains of SARS-CoV, SARS-CoV-2, and MERS 9CoV are orange, dark blue, and green, respectively. Variable residues between SARS-CoV and 2019-nCoV in the receptor-binding site are highlighted as sticks. CoV =. 2019-nCoV = 2019 novel. SARS-CoV-2 = severe acute respiratory syndrome. MERS = Middle East respiratory syndrome (Reproduced with permission from publisher from reference, Lu et al. 2020, Lancet. 2020 Feb 22;395(10224):565-574) [4].

The phylogenetic analysis of COVID-19 with other reported viruses indicated SARS-CoV-2 has a small genetic distance from the bat, a 40–90% similarity with SARS-CoV, and 20–26% similarity MERS-CoV. Also, the phylogenetic analysis of SARS-CoV-2 with related genera and representatives of beta-coronaviruses had shown that the five subgenera and five branches were formed. It has been envied that the receptor-binding domain of beta-coronaviruses located in the C-terminal domain of S1, noted for SARS-CoV29 for lineage B, and MERS-CoV and BatCoV HKU, as seen in the lineage C (Figures 2 and 3). The SARS-CoV-2 (COVID-19) has S-protein strong interaction with human angiotensin-converting enzyme-2 (ACE2) molecules, which interacts with *S-protein-ACE2* pathway. This led to the further implication of this virus transmission in humans posing severe public health risks and modeled through virus spike protein [37]. Deng and Peng (2020) mentioned the guidelines for the treatment of COVID-19 should be followed for general and specialized procedures along with the responsibility of different organizations in the outbreak. This led to entirely or partially lock-down and of approximately 60 million affected people and local transportation in Beijing and Shanghai. Similar, instances had occurred in other countries by which human contact restriction, contact with wild animals, airport screening, creation of isolation centers, cancellation of flights, new isolation wards and new medical units constructed, etc. Instead of person- to- person respiratory and body contact, the fecal-oral transmission also acts as one of the routes of infection of this deadly virus [38]. The case of tertiary transmission of the case of an indexed patient in Korea through the use of Lopinavir/Ritonavir for COVID-19 treatment, detection thorough RT-PCR [25] and other clinical laboratory features determined through imaging method [39].

## 4. Precautions to fight against coronavirus

Recent transmission of Ebola virus, Nipah virus, MERS, and Crimean-Congo hemorrhagic fever, have indicated the risk of nosocomial transmission of emerging infections in healthcare settings [20]. SARS and MERScan persist to inanimate surfaces (2–10 days) and can be inactivated by biocidal agents [40]. However, there is no specific therapy available for SARS-CoV-2 initial containment and prevention of spread [40]. Therefore, COVID-19 is a rapidly evolving situation and new, even stable for hours on the surfaces similar to other SARS virus [41]. As per the rapid advance guidelines for diagnosis and treatment of SARS-CoV-2 infected *Pneumonia* (standard version), these guidelines are first to hospital staff and medical professionals, community residents, and public health persons and researchers as well [42]. For the precaution point of view, the specific guidelines to be followed, available from (www.coronavirusgov and http://who.int, WHO guidelines). We need to watch for symptoms to appear in the patient with travel history or human-to-human contact in the past few days. The illnesses vary from mild to severe illness symptoms, and even death occurs among the COVID-19 in severe cases and the standard guidelines for testing of COVID-19 should be followed (www.coronavirus.gov). The COVID-19 clinical toolkit, diagnosis guidelines and analysis toolkit, available from:(https://www.elsevier.com/clinical-solutions/covid-19-toolkit). In addition to this-WHO, CDC and FDA guidelines should be followed for the new diagnosis, prevention, and treatment options.

## 5. Management and treatment options

The use of vaccines and/other therapeutics for the treatment of novel COVID-19 is the urgent need of today, but it takes time to reach this goal. Today, various labs around the world are continually working to find out the solution for the treatment of this deadly disease, to combat the high mortality and further warn of epidemics. WHO has classified this disease as a pandemic with a possible cure through various medical management strategies. Therefore, the novel coronavirus is putting the whole world on alert [1], and there is an urgent need to maintain regular physical activity and boost the immune system against coronavirus contagious respiratory disease [36]. Zhang and Liu, proposed potential interventions for COVID-19 based on the previous treatments for SARS and MERS [43]. The general treatment includes nutritional interventions like Vitamin A, B, C, D, E, polyunsaturated fatty acids (PUFA), selenium, zinc, iron; immunomodulators (interferons, gamma globulins, thymosin alpha-1, levamisole, cyclosporin); antiviral treatments (ribavirin, lopinavir,(LPV)/ritonavir (RTV) (Kaletra), remdesivir, nelfinavir, arbidol, nitric oxide): other compounds (alpha-lipoic acid, estradiol, and phytoestrogen, mucoporin-M1) [43]. It has been observed that extensive treatment helps to boost up the immune response, and later advanced treatment methods control the virus at the advanced infection stage. The case studies have shown that maternal to child transmission of SARS-CoV-2 does not occur as per date with available reports. Therefore, we need to take the utmost care in the research and need to develop the methods to combat such emerging infections in the future. But, this has left some questions unanswered, as here, in this case, animal to human transmission of COVID-19 disease and among plant-animal-human-microorganisms or other species disease(s) and vice-versa transmission can be possible?

### 5.1. Activating innate and adaptive immunity

Innate and adaptive immunity plays a vital role in combating the viral/bacterial pathogenesis and clearing off the infection. In this context, various co-stimulatory molecules are in the pre-clinical or clinical trial and have shown tremendous efficacy. One such molecule, SA-4-1BBL (an agonist of the natural 4-1BB receptor), have been shown to promote Th1 type of innate and adaptive immune responses, with the production of a plethora of effectors cytokines like IFN-γ, TNF-α, and IL-2. SA-4-1BBL have been shown to protect against multiple cancer models [44]–[46] and bubonic/pneumonic plague mouse models [47],[48] and thus can be targeted for the treatment of immune-compromised COVID-19 patients.

### 5.2. Passive serum transfer and monoclonal antibodies

Passive serum transfer from the recovered patient to the newly infected patient has known for the last 100 years and used to decrease the disease severity recently during the 2009 H1N1 pandemic and 2013 Ebola epidemic. Casadevall et al., have shown that passive serum transfer therapy could be used for the treatment for COVID-19 [49]. A recent study on rhesus macaques clearly indicated that antibodies have a tendency to prevent secondary re-infection with SARS-CoV-2 [50]. Based on these studies, FDA has approved the passive plasma transfer from COVID-19 survivors to seriously ill patients under an emergency investigational new drug (IND) protocol. Grifoni et. al, have measured the T cell response from 10 COVID-19 cases and 11 healthy controls and observed that recovered patients generated significant CD4^+^ T cell response against SARS-CoV-2 with polarization to classical Th1 phenotype (IL-2 and IFN-γ), whereas low or no IL-4, IL-5, IL-13 and IL-17 [51].

Even-though, isolation of plasma from patients is difficult in terms of quantity and quality but its active component (neutralizing antibodies) can be isolated easily and scale up for mass production. Several groups have isolated and purified mAbs against SARS-CoV and MERS-CoV using different techniques such as phage display or from plasma of infected patients [52],[53]. Wang et al., has similarly synthesized human 47D11 H2L2 mAb, that effectively inhibit the SARS-CoV, SARS-CoV-2 and MERS-CoV infection in VeroE6 cells [54]. Pinto et al., have identified multiple monoclonal antibodies from memory B cell population from SARS-CoV (2003) patient and identified S309 human monoclonal antibody, that shows potent neutralization of SARS-CoV-2 and SARS-CoV pseudoviruses [55].

### 5.3. Vaccination against coronavirus

Developing treatment against SARS-CoV-2 will likely be useful for long-term protection, as it mutates slowly than the flu virus, so there is an urgent need for developing the vaccine. While live attenuated vaccines targeting respiratory viruses, including influenza viruses and adenoviruses, have been approved for use in humans, but these vaccines have the chance to revert back to the virulent strain [56] or recombination with parental/other strains [57]. Elaine et al., have shown the efficacy of live attenuated strain lacking E-gene against SARS-CoV-1 infections in syrian golden hamsters [58]. Codagenix, in collaboration with Serum Institute of India, has created a live-attenuated SARS-CoV-2 vaccine by changing the optimized codons with non-optimized codons with large-scale manufacturing [59].

A step forward in this direction, different therapeutic and vaccine designing approaches against coronaviruses are being explored and will be evaluated in terms of their potency, efficacy and safety and available in future soon [60]–[62]. Protein based subunit vaccines are most efficacious, but these are dependent on adjuvants for their immunogenicity. Among all structural proteins of SARS-CoV, S-protein is mainly responsible for inducing host immune responses and protective immunity against virus infection with neutralizing-antibodies and T-cell responses. Thus, full-length S-proteins are believed to be the most critical target for CoV-2 vaccine development [63],[64]. Tai et al. have identified receptor-binding domain (RBD) in SARS-CoV-2 S-protein, with a very high affinity for human and bat angiotensin-converting enzyme 2 (ACE2) receptors and thus inhibiting the infection to host. RBD based vaccines can be of high potential for the prevention of SARS-CoV-2 [65]. Novavax has designed virus-like nanoparticles (VLPs) based on SARS-CoV-2 S-protein [66]. Clover Biopharmaceuticals developed a trimerized SARS-CoV-2 S-protein subunit vaccine using their patented Trimer-Tag® technology [66]. Baylor College of Medicine have shown the efficacy of SARS-CoV RBD vaccine with alum elicits high levels of humoral and cellular response and protective immunity upon virus challenge with minimal host immunopotentiation [66].

Other than these, several biotech companies are developing other novel vaccine approaches using DNA vaccines. Yu et al. has evaluated the efficacy a DNA vaccine expressing different forms ofSARS-CoV-2 Spike (S) protein in rhesus macaques and shown the complete protection with decreased median viral loads in lavage and nasal mucosafollowing SARS-CoV-2 challenge [67]. A vaccine based on full length S protein of SARS-CoV-2, lipid nanoparticle (LNP)-encapsulated mRNA-1273 has shown positive interim phase I data and now moved to pphase II/III clinical trial to assess the safety, reactogenicity, and immunogenicity, (available from: https://clinicaltrials.gov/ct2/show/NCT04283461). Several other mRNA-based vaccines such as CureVac (Tübingen, Germany), BNT162 by BioNTech (Mainz, Germany) are in pre-clinical testing [68]. Inovio Pharmaceuticals' INO-4800 is a MERS Coronavirusspike gene DNA-based vaccine, has evaluated in mice and guinea pigsand has already entered phase I clinical trials [68]. Oxford University, UK and AstraZeneca has developed ChAdOx1 SARS-CoV-2 (weakened common cold adenovirus vector) and has shown the efficacy in phase I clinical trial and now moved to phase II/III of the clinical trial [69].

### 5.4. Antiviral compounds

Currently, there is no much information available on protein structure and function for SARS-CoV-2, and hence there are no effective antiviral agents for the treatment of patients. Giri et al. (2020) has shown the similarity pattern between the Human SARS and bat SARS and observed an array of disordered proteins like Nucleocapsid, Nsp8, and ORF6 in SARS-CoV2, which can be of particular importance for potential drug targets [70]. Zhang etal. deciphered the 3D structure of the SARS-CoV-2 main protease with high-intensity X-rays synchrotron and observed α-ketoamide 13b inhibitor has the tendency to target the main protease which releases 12 smaller proteins for viral replication machinery. The α-ketoamides can be potential drug targets as they have direct delivery to the lungs[71],[72]. Gordon et al., has cloned 26 viral proteins from SARS-CoV-2 and revealed the 332 numbers of high confidence in human protein-protein interactions. Out of 332, 67 FDA approved druggable antiviral targets are currently in evaluation for efficacy against live SARS-CoV-2 infection assays [73].

The broad-spectrum antiviral compound Griffithsin has been shown to prevent Nipah virus in Syrian Golden Hamsters [74], HIV-1, HSV-2 [76], MERS [77] and other enveloped virus [78], as virus entry inhibitor and has been shown efficacious in non-human primates against HIV [79],[80] and this act as outstanding antiviral Lectin having therapeutic potential[75]. Moreover, Griffithsin (GRFT) has been shown to prevent SARS-CoV infection both in vitro and in vivo [81]. Griffithsin could eventually lead to a therapeutic agent against COVID-19. Loutfy et al., in a preliminary study, observed the improved oxygen saturation and reduced lung abnormalities withInterferon *Alfacon-1* and corticosteroids in severe acute respiratory syndrome (SARS) patients [82]. Chu et al. indicated the role of lopinavir/ ritonavir for the treatment of SARS [83]. The use of Baricitinib (Olumiant), a drug for the treatment of rheumatoid arthritis, envied as the potential treatment option of SARS-CoV-2 disease [84].

## 6. Potential future treatments

Experts of opinion by Smith and Posser, (from Pharm D), available from: https://www.elsevier.com/connect/-information-center, are reproduced here to understand the new development for the treatment of COVID-19 *‘for the potential drug therapy and targets. Ribavirin and interferon were not recommended as first-line treatments because of the risk for side effects; however, the use of these medications may be considered if treatment with lopinavir; ritonavir, chloroquine, or hydroxychloroquine are ineffective’* [11].

Information available from the Bloomberg website, (www.bloomberg.com), indicated that *‘Remdesivir (GS-5734), a nucleoside analog: inhibits the activity of 2002 SARS-CoV, MERS-CoV, and bat CoV strains that can replicate in human epithelial cells and mediate entry via human CoV receptors. This has been administered to several hundred patients with confirmed, severe SARS-CoV-2 infections in the United States, Europe, and Japan through Expanded Access or Compassionate Use programs (available from: Bloomberg website,* www.bloomberg.com) and *drug manufacturer (Gilead Science, Inc.* available from: http://gilead.com)’.

- Another nucleoside analog, EIDD-2801 blocks RNA polymerase for viral assembly in a similar manner to Remdesivir, is in phase III clinical trial. Remdesivir can only be given intravenously; in contrast, EIDD-2801 can be provided in pill form [85],[86].
- The research in the identification and development of monoclonal antibodies specific to SARS-CoV-2 are still in progress. Therefore, the combination therapy: SARS-CoV-2 specific monoclonal antibodies with Remdesivir can be the ideal therapeutic option [87]. Regeneron is trying to identify mAbs specific and effective for COVID-19. But, the evaluation of efficacy is required for confirming this novel combination therapy.
- Wang et al (2020) suggested that *‘In preclinical trials, remdesivir has demonstrated significant activity against and a high genetic barrier toresistance.*
- Elfiky (2020) suggested the use of ‘*Sofosbuvir in combination with ribavirin: Data from a molecular docking experiment using the SARS-CoV-2 RNA dependent RNA polymerase (RdRp) model identified tight binding of sofosbuvir and ribavirin to the RdRp, thereby suggesting possible efficacy of sofosbuvir and ribavirin in treating the COVID-19 infection*’ [88]. Further data will continue to emerge regarding antiviral therapy for SARS-CoV-2.
- In a study by Malik et al (2018) described the role of a DNA aptamer AS1411 as potential treatment option for several respiratory viruses' infection, taking advantage of cell surface protein ‘nucleolin’, many viruses bind to it for translocation into the cells, whereas AS1411 inhibit the entry of viruses to prevent infection including SARS-CoV-2 (unpublished data). However, their exact mechanism is unknown [89].

## 7. Conclusions

In view, the present outbreak of coronavirus and other similar past diseases like SARS, MERS, etc. This is an alarming situation when we have seen such a massive spread on coronavirus infection through interstate, national, and international boundaries. What should be the base for intra and interspecies movement of the pathogenic microorganism? In the review, by Vincent et al., (2007), the authors indicated that like SARS and MERS might undergo the genetic mutation and recombination to convert into more virulent form. In this outbreak, medical and scientists have shown through their constant efforts, that the strain characterized using new sequencing platforms and instantly shared to the public domains (Table 1). The fast sharing of literature and research soon helped us design the vaccine for such pandemic infection, and in the coming few months, we may have a vaccine for this disease. The molecular, genetic, and immunological methods indicated the disease prognosis and transmission through one organism to another. Sequencing and bioinformatics approaches led to finding similarity of this virus with another deciphered virus. The animal-like bats, cats, camel, and horseshoe bats act as a reservoir of the virus, which might lead to the emergence of new re-emerging infection [90]. The treatment options include antivirals, therapeutic antibodies, cytokines, nucleic-acid based therapies to target viral gene expression and vaccines. Giri et al. (2020) has shown the similarity pattern between the Human SARS and Bat SARS and observed an array of disordered proteins like Nucleocapsid, Nsp8, and ORF6 in SARS-CoV2, which can be of particular importance to structural biologists for potential drug targets [58]. The use of an Artificial Intelligence (AI) based algorithm by Benevolent AI gives the vital information of molecules in medical science. More recently, this method has been used to screen the novel antibiotics, Helicin from a series of compounds tested through AI and deep learning-based algorithms based discovery of molecules [91].
